# Case Report: Off-Label Use of Omalizumab in a 6-Year-Old Boy With ASD Ameliorated Severe Allergic Rhinitis and Subsequently Improved Behavioral Symptoms

**DOI:** 10.3389/fped.2021.714111

**Published:** 2021-09-22

**Authors:** Xue-Jun Kong, Cullen Clairmont, Bryan Wang

**Affiliations:** ^1^Martinos Center, Massachusetts General Hospital, Boston, MA, United States; ^2^Department of Medicine, Beth Israel Deaconess Medical Center, Boston, MA, United States

**Keywords:** allergic rhinitis, autism spectrum disorder, sleep disturbance, anxiety, omalizumab

## Abstract

Children with ASD have elevated risk for developing allergic symptoms. The severity of allergic symptoms can exacerbate behavioral problems in children with ASD. Omalizumab, an anti-IgE antibody, has previously shown efficacy in treating allergic rhinitis and behavioral problems in a 12-year-old child with ASD. The present case report provides robust characterization of behavioral improvement in a 6-year-old child with ASD, allergic rhinitis, and autoimmune disorder. A 6-year-old boy with ASD and Hashimoto's disease presented to the clinic with severe allergic rhinitis, irritability, and language delay. After other treatments failed to improve symptoms, our patient was treated with omalizumab at 300 mg/month via subcutaneous injection for a total of 6 months. Marked improvement in allergic symptoms were observed at 2 months into treatment and were maintained through the treatment period. At the conclusion of the treatment period, results from multiple behavioral questionnaires, including the SRS-2, ABC, RBS-R, and PSQI, demonstrated substantial improvement in ASD-related behavioral symptoms. In this case, omalizumab markedly improved ASD-related and sleep behavior in a 6-year-old with ASD, allergic rhinitis, and autoimmune disorder. Future studies with larger patient populations are warranted to investigate the efficacy of omalizumab in patients with ASD and allergy symptoms.

## Background

Autism Spectrum Disorder (ASD) is a complex neurodevelopmental disorder characterized by social communication deficit and repetitive behaviors which has become increasingly prevalent over the last several decades. According to the latest CDC reports, the prevalence of autism has risen to 1 in 54 children (1); by 8 years old, nearly two percent of children are diagnosed with ASD. ASD often presents with a wide-variety of behavioral problems, including sleep pattern abnormalities (2, 3) and anxiety (4, 5). Therapies that ameliorate these comorbid symptoms of ASD and improve quality of life are highly desirable.

The potential epidemiological and pathological link between ASD and allergic disease (6, 7) may provide a new therapeutic avenue for the subpopulation of children with ASD and allergic symptoms. This subpopulation is considerable, given the high co-occurrence rate of ASD and allergies (8). A case control study of children and young patients with ASD (*n* = 5,565) and controls (*n* = 27,825) matching in birth year and sex reported that allergies, asthma, and autoimmune disorders were diagnosed more frequently in ASD patients than controls (9). Notably, a significant positive correlation between the frequency of allergic symptoms and ASD severity has been reported (10). Many patients with allergies, including those who are also diagnosed with ASD, have elevated circulating IgE levels (11). IgE mediates the activation of mast cells, which release a cell-specific set of inflammatory molecules and initiates the allergic reaction cascade. Mast cell activation has also been implicated blood-brain barrier maintenance (12) and neuroinflammation (13, 14). We hypothesize that the pathophysiology of IgE-mediated allergies may exacerbate behavioral symptoms in children with ASD.

**Figure 1:**
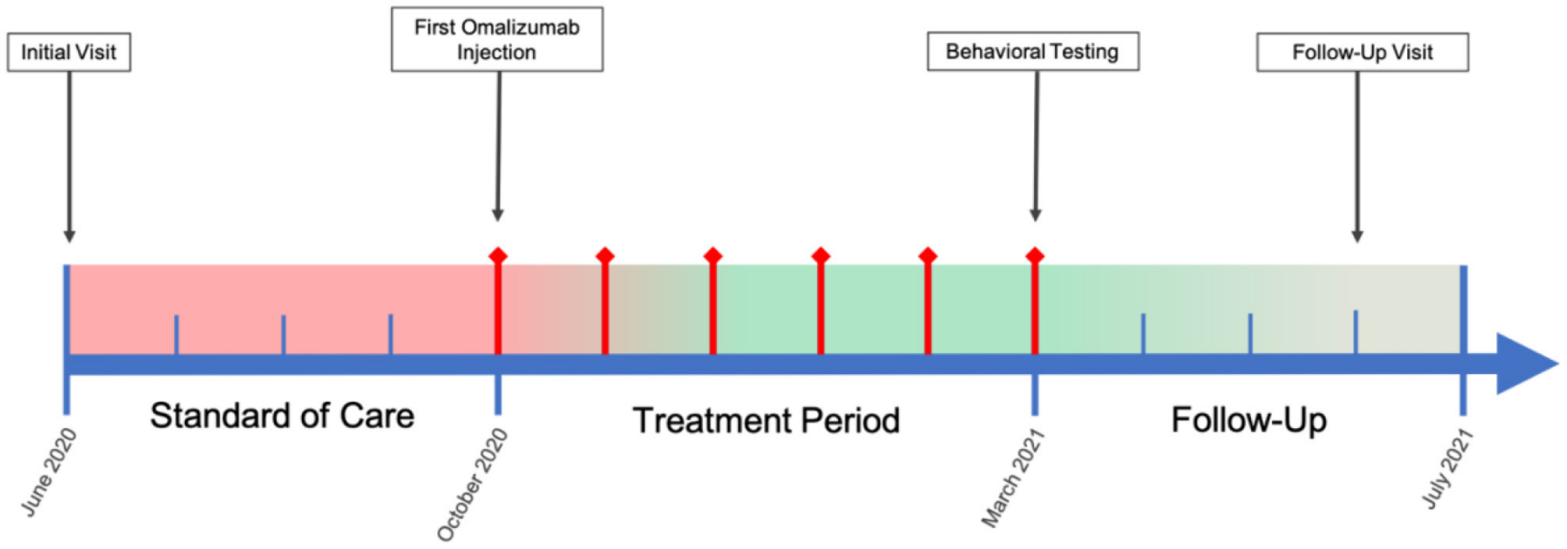
Case Timeline. Color bar is a qualitative, visual representation of patient overall symptom severity over the course of the case, wherein red indicates severe symptomology and green mild/no symptomology. Red diamond timepoints indicate months during which omalizumab treatment was administered.

In the present case study, we investigated the potential efficacy of an immunological intervention in a pediatric patient with ASD and comorbid severe allergic rhinitis and Hashimoto's disease. It is well-reported that both IgE-mediated mast cell activation and autoimmunity are potential pathogenic factors for ASD (13, 15). Humanized murine anti-IgE antibody (omalizumab) may have value in the treatment of ASD in patients who have comorbid allergies and elevated IgE levels. The binding of omalizumab to IgE inhibits the attachment of IgE to its receptor, FcεRI, on mast cells and has been found to reduce surface IgE receptor levels and the ability of these cells to be activated by allergens (16). Omalizumab is approved for the treatment of asthma, idiopathic urticaria, and nasal polyps and has shown robust efficacy in the resolution of allergic symptoms and improvement of quality of life (17–19). In 2015, Jyonouchi published a case report, which showed that the use of omalizumab significantly improved neuropsychiatric symptoms of two children with severely limited expressive language, one of whom was diagnosed with ASD, and allergic rhinitis (20). Since then, no other report using omalizumab in this subpopulation has been published. Here we present another interesting case of a 6-year-old boy with ASD and comorbid autoimmune conditions, including Hashimoto's disease and severe allergic rhinitis, being treated with omalizumab. We employed several more highly relevant outcome measures than the previous case report did: The Social Responsiveness Scale (SRS-2), Repetitive Behavior Scale-Revised (RBS-R), and Aberrant Behavior Checklist (ABC) were used to assess ASD core symptoms; the Epworth Sleepiness Scale (ESS) and Pittsburgh Sleep Quality Index (PSQI) were used to assess sleep quality and disturbance; the Generalized Anxiety Disorder 7-item (GAD-7) scale was used to assess general anxiety; the Rhinitis Control Assessment Test (RCAT) was used to assess allergic rhinitis control status; and the Clinical Global Impressions (CGI) Scale, including CGI-Severity (CGI-S) and CGI-improvement (CGI-I), was used by a clinician to assess global improvement. See Figure 1 for case timeline.

## Case Presentation

A 6-year-old Asian boy, who was a full-term vaginal birth with an Apgar score of 10, was diagnosed with developmental delay and ASD. He met the motor milestones of sitting, standing, and walking at the appropriates ages. He spoke his first word at 7 months; however, he lost language capabilities at 8 months and exhibited less frequent eye contact. At 18 months, he stopped responding to his name and making eye contact at all. At the age of 2 years and 9 months, he was diagnosed with acute disseminated encephalomyelitis (ADEM) and received IV steroid treatment, which resulted in significant language improvement. He started using a few words and echoing. His motor function was also improved, as he was now able to jump with both feet off of the ground and could utilize more fine motor functions. Overall, he remained developmentally delayed. At 5 years of age, his attending physician confirmed significant general developmental delay and ASD diagnosis. Probiotics and nutritional support failed to elicit any significant improvement. He was diagnosed with Hashimoto's disease; his mother, during her pregnancy with him, was also diagnosed with Hashimoto's disease and was taking levothyroxine throughout the whole pregnancy.

At the time of his initial visit in June of 2020, he was found to be thyroperoxidase (TPO) and anti-thyroglobulin (anti-Tg) positive with elevated thyroid-stimulating hormone (TSH) at 8.9 (0.56–5.9), while his free T4 and total T3 were normal. He was also found to have elevated IgE (>300 IU/ml), averaging about 600 IU/ml across three different blood samples. He was positive for folic acid receptor antibody both binding antibody (85.8) and blocking antibody (185.90). He was found to be severely allergic to dust mites (4+) and pollen (4+) via allergy testing, and he suffered from severe hay fever with frequent to daily rhinorrhea, sneezing, and coughing. He was diagnosed with allergic rhinitis and angioedema but had no diagnosis of asthma or urticaria. He experienced seasonal exacerbation in the spring, during which time he presented with worsened eyelid swelling, tearing, rhinorrhea, and difficulty sleeping, none of which had resolved with antihistamine, nasal steroid, and Singulair treatment in the past. He had dark circles and swelling around his eyes, severe nasal congestion, conjunctiva congestion, and itchiness all over, especially in the genitalia area, but had no rash. He had abdominal distension and constipation. His language was delayed, and he exhibited problems with articulation. He exhibited self-stimming and poor attention, and avoided eye contact and social interactions. He was easily irritated and emotionally sensitive, often becoming angry, upset, or anxious. Despite normal MRI and EEG, he had been experiencing poor sleep quality, including difficulty falling to sleep and frequent sleep disturbance with crying. He was treated with Claritin 5 mg/day, Singulair 5 mg/day, Cromolyn sodium 100 mg three times per day, Ranitidine 40 mg twice per day, and low dose naltrexone 6 mg per day topically. After 4 months (October 2020), his TSH was reduced to 5.64 (within the normal range of 0.56–5.9), while his IgE remained elevated at 695 IU/ml. Clinically, he exhibited minimal changes: he still had eyelid redness and swelling at times, severe nasal congestion, rhinorrhea, and frequent sneezing; his irritability, labile emotion, anxiety, and sleep disturbance were unchanged.

**Figure 2:**
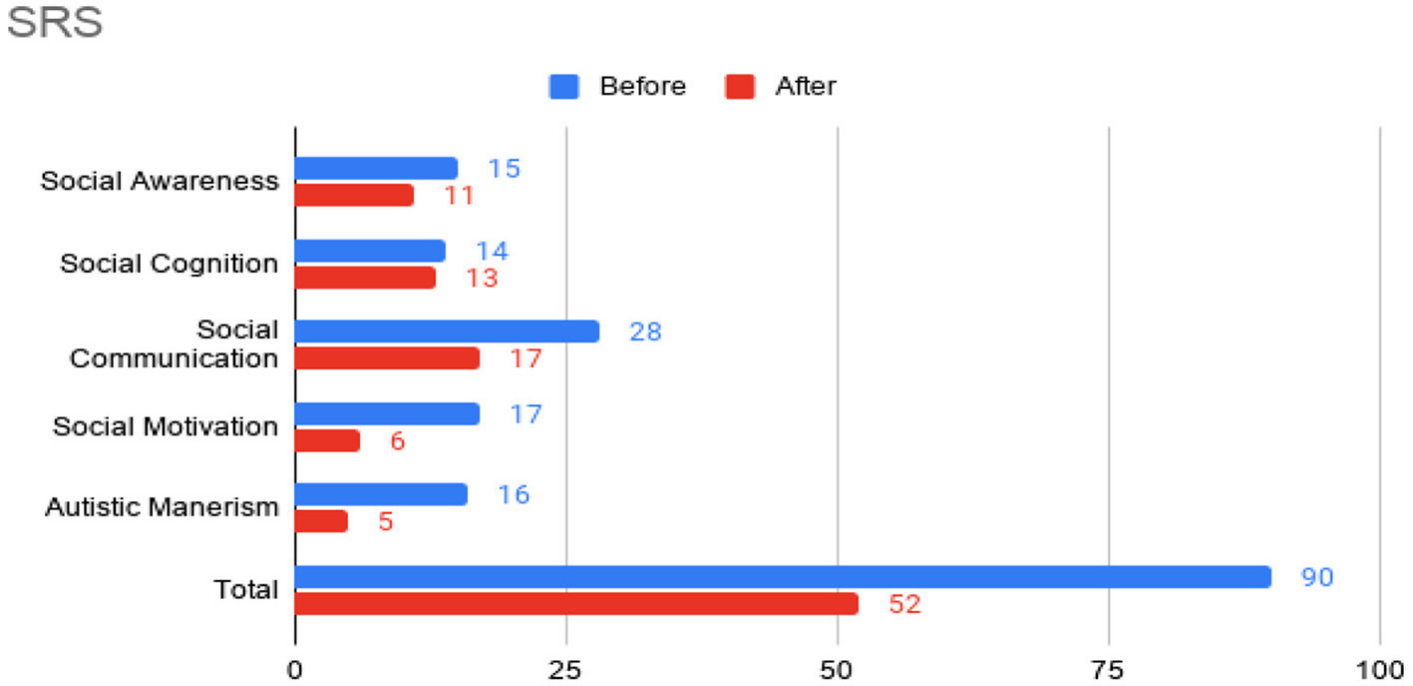
Social Responsiveness Scale-2 (SRS) sub-scores prior to the initiation of omalizumab (blue) and after the 6 month treatment period (red).

**Figure 3:**
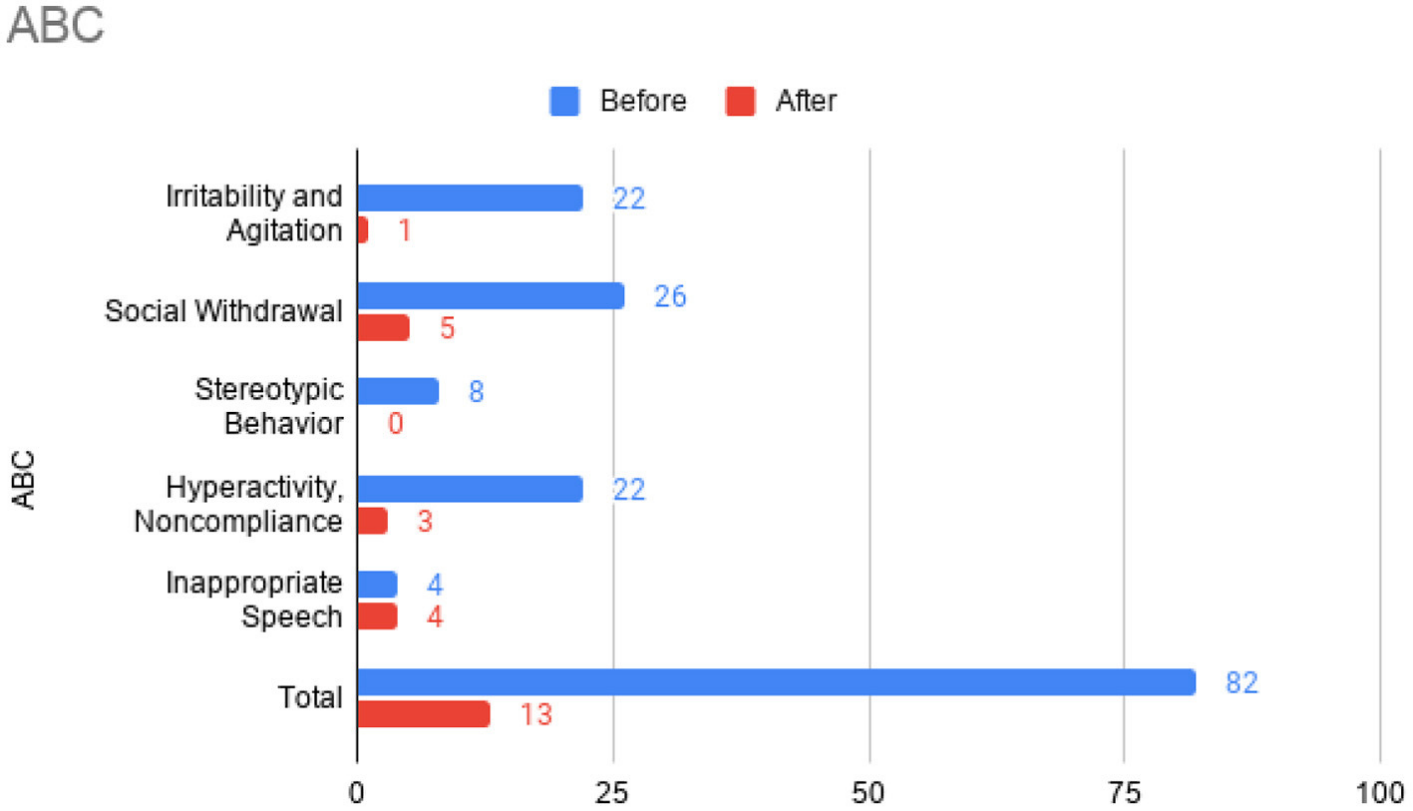
Aberrant Behavior Checklist (ABC) sub-scores prior to the initiation of omalizumab (blue) and after the 6 month treatment period (red).

**Figure 4:**
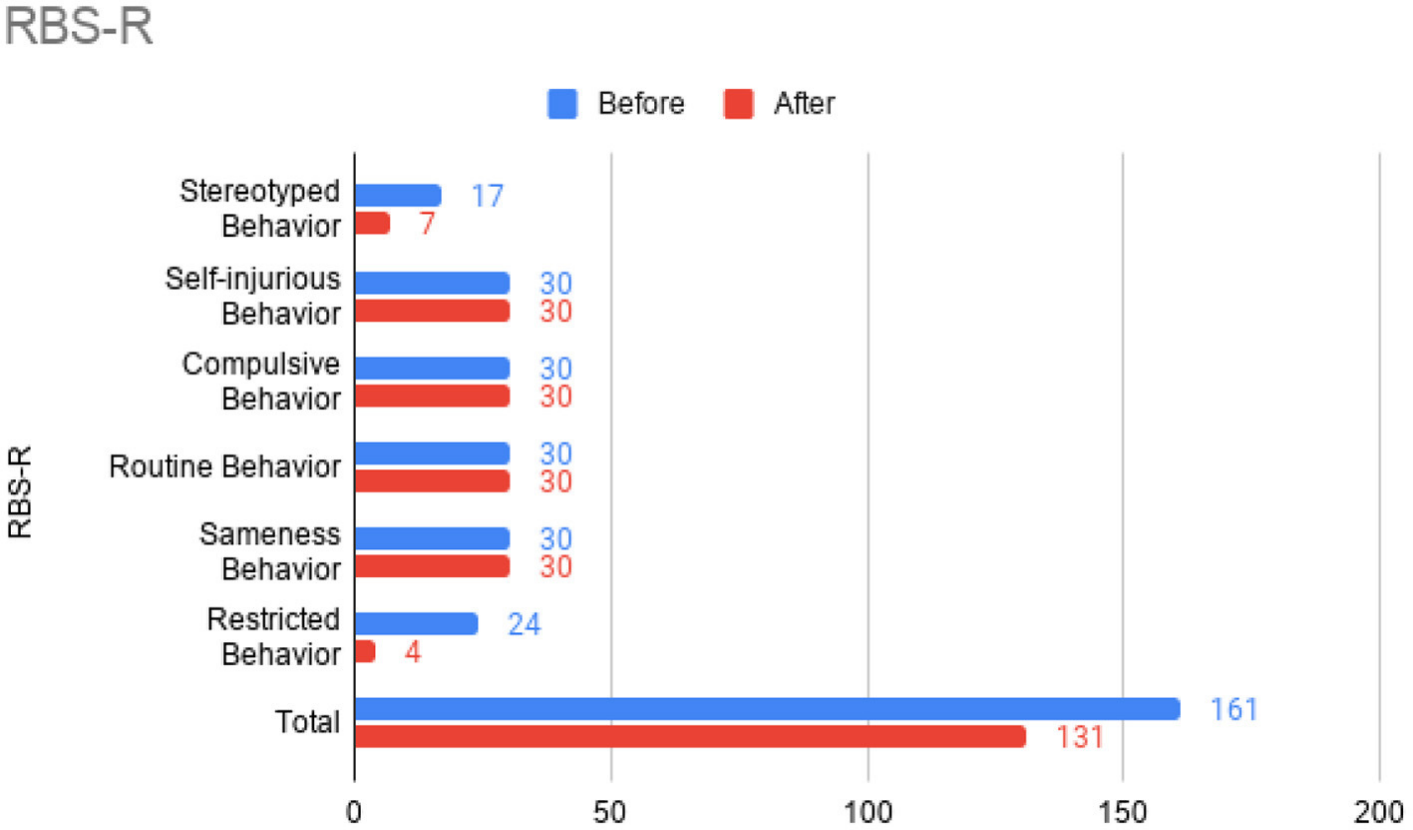
Repetitive Behavior Scale-Revised (RBS-R) sub-scores prior to the initiation of omalizumab (blue) and after the 6 month treatment period (red).

**Figure 5:**
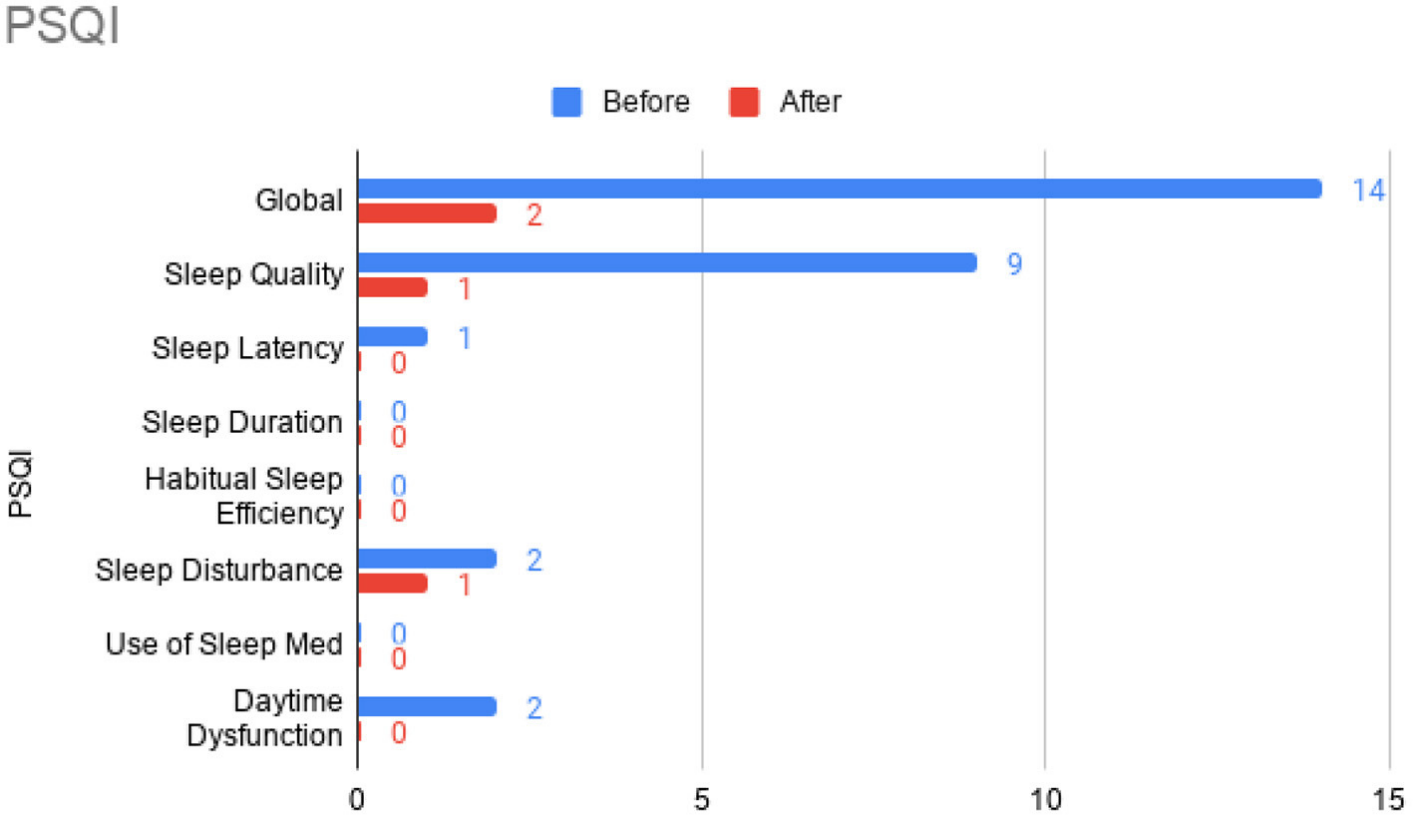
Pittsburgh Sleep Quality Index (PSQI) sub-scores prior to the initiation of omalizumab (blue) and after the 6 month treatment period (red).

**Figure 6:**
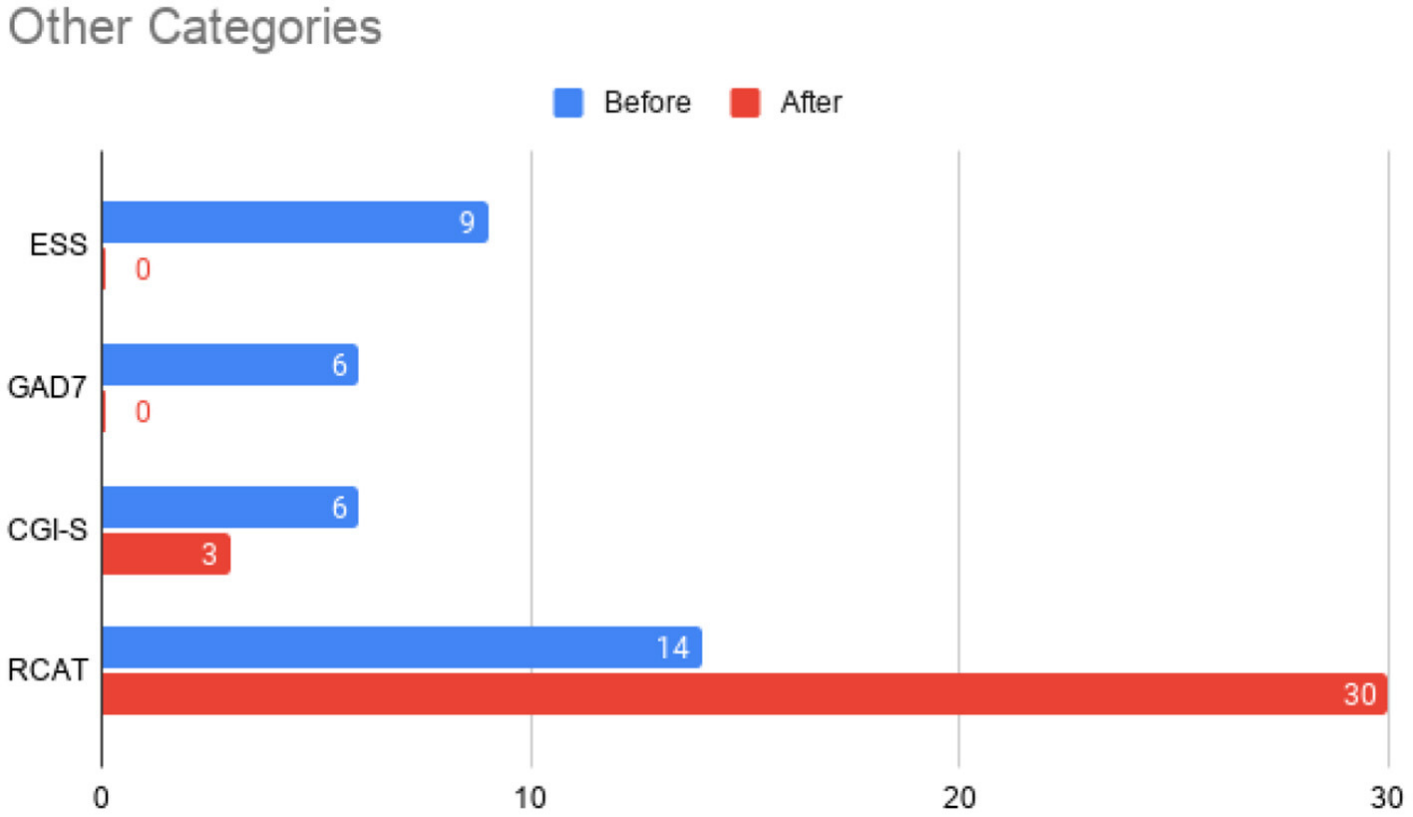
Total scores before initiation of omalizumab (blue) and after the treatment period (red) for the Epworth Sleepiness Scale (ESS), General Anxiety Disorder 7-item (GAD-7) scale, Clinical Global Impressions – Severity (CGI-S) scale, and the Rhinitis Control Assessment Test (RCAT). In contrast to other parameters, RCAT scores indicate better improvement with higher scores, while the rest of the tests indicate better improvement with lower scores. The Clinical Global Impressions - Improvement (CGI-I) was rated a 2 at the conclusion of the treatment period, indicating “much improved” symptoms.

At this time, he was administered omalizumab at 300 mg per month via subcutaneous injection (Novartis, 150 mg per vial, two injections at two injection sites for each dose) for a total of 6 doses over 6 months. His body weight and IgE level were used to calculate dosing regimen, according to the FDA-approved omalizumab dosing chart for allergic asthma patients between 6 and 11 years old. The patient received injections in a local hospital, where he was observed for 2 h after each injection. His allergic symptoms were noticeably improved after 1 dose and continued to improve; marked improvement was observed at 2 months of treatment. He denied itchiness, had controlled nasal symptoms, and was much less irritable. He also had improved cognitive function after the second dose; for example, he could add and subtract accurately with digits below 20 (before treatment, he could not reliably recognize single digit numbers). After the second dose, his behavioral symptoms, including his social communication and social receptivity, were noticeably improved, based on parent reporting and clinical observation. At the conclusion of the treatment period, we used several questionnaires to compare pre- and post- treatment severity of ASD-related symptoms, sleep disturbance, anxiety, and allergic rhinitis. Over the course of the 6 month treatment period, he tolerated the drug well: He reported no major or minor adverse effects, including but not limited to anaphylaxis, headache, dizziness, fatigue, skin rash and itchiness, bone fractures, pain or discomfort of the ears, fever, cough, sore throat, or any other discomfort. To better evaluate the efficacy of this treatment, the caregiver (his mother) was asked to complete the following questionnaires: SRS-2, RBS-R, ABC, ESS, PSQI, GAD7, RCAT before and after 6 month treatment. CGI was evaluated by the treating clinician and interview with the mother. These measurements demonstrated substantial improvement in ASD-related behavioral symptoms, sleep quality and sleep disturbance, anxiety level, and allergic rhinitis symptoms. These results are shown in Figure 2 (SRS-2), Figure 3 (RBS-R), Figure 4 (ABC), Figure 5 (PSQI), and Figure 6 (ESS, GAD7, RCAT, and CGI).

Omalizumab was interrupted after 6 months in March 2021. During a follow-up visit in June of 2021, some relapse of allergy symptoms were noted. However, the symptoms of hay fever were mild, brief, and easily controlled by antihistamine use with no known recurrence of symptoms to date. His parent rated his allergic symptoms 80–90% better than in previous years during allergy season. His parent also rated his mood, anxiety, and sleep quality at 80–90% improved. His parent estimated that repetitive behaviors such as repeatedly pressing buttons, wandering, and hand flapping were reduced by 50%. His parent confirmed sustained improvement in social awareness, social communication, and cognition. His parent noted that his language improvement was gradual during the treatment course but was most noticeable 1 month after the conclusion of the treatment period (April 2021) and sustained throughout the rest of the follow-up period: his ability to use active and descriptive language was markedly improved. Overall, his parents strongly believe that omalizumab resulted in sustained benefit for their son.

## Discussion

The present study shows substantial improvement of allergic rhinitis, ASD-related behavioral symptoms, sleep, anxiety, and overall CGI score in a 6-year-old boy during and after 6 months of treatment with omalizumab. This case presentation illustrated that allergies can exacerbate ASD-related behavioral problems. Omalizumab may be a promising symptomatic treatment for a subgroup of ASD individuals with significant IgE-mediated allergic illness, most commonly presenting with allergic rhinitis. This case is a representative example in this subgroup, which accounts for about one fifth of the whole ASD population (9).

The use of omalizumab, a humanized monoclonal antibody able to bind to IgE, is currently approved only for treatment of severe bronchial asthma, idiopathic urticaria, and nasal polyps. The use of omalizumab in other IgE-related diseases, such as allergic rhinitis, is off-label. The efficacy of omalizumab in treating allergic rhinitis (21, 22)or rhinitis with nasal polyps (23) has been previously reported. A pilot study showed that nasal obstruction, rhinorrhea, itching, and sneezing were significantly reduced (22). Turbinate hypertrophy was resolved in six of nine patients, and eight of eleven patients (73%) had their allergic rhinitis reduced or eliminated (22). The clinical observation and improvement in RCAT score reported in this case further supports the growing evidence for the efficacy of omalizumab in uncontrolled allergic rhinitis.

The potential therapeutic effect of omalizumab in children with developmental delay and poorly controlled allergic rhinitis was investigated in a single, previous case report by Jyonouchi in 2015, which reported marked improvement of neuropsychiatric symptoms, such as hyperactivity and anxiety, in two 12-year-old patients (20). ABC total score and all sub-scores were improved after the treatment of the patient who was also diagnosed with ASD. No other case report with this indication has been published. In the present case report, we administered several questionnaires (including ABC) before and after treatment to robustly demonstrate changes in ASD-related behavior. Across these questionnaires, we also found substantial improvement in social function, restricted and repetitive behaviors, hyperactivity, anxiety, and sleep disturbance after omalizumab treatment. Like Jyonouchi, we found marked improvement of allergic rhinitis within a month after the first injection and subsequent improvement of neuropsychiatric symptoms. Specifically, we began to observe improved cognition and ASD-related behavior after two injections over 2 months. The resolution of allergic symptoms preceding improvement of neuropsychiatric symptoms indicates the role allergies may have in exacerbating cognitive and behavioral symptoms in children with ASD. Future work that illuminates the mechanisms underlying this exacerbation would be highly valuable. While the discomfort caused by allergies may be the simple explanation, the previously found associations between ASD and allergies (8–11) and the implications of mast cell overactivation in neuroinflammation (12–14) indicate a more complicated pathophysiology.

Notably, the present case study reports positive effect of omalizumab in a 6-year-old; Jyonouchi reported findings at the age of 12. In 2016, omalizumab was approved for people between 6 and 11 years of age with persistent asthma in the United States; previously, omalizumab was only approved for treatment of people 12 years and older. We found that the 6-year-old in this case tolerated a 300 mg monthly injection well-without any adverse effects. In this case, we found that omalizumab treatment of a 6-year-old boy with ASD and autoimmune disorder was safe and efficacious. His response timeframe closely followed that of the 12-year-old boy reported in Jyonouchi, even though our patient, in addition to being younger, had other types of autoimmunity (positive TPO, anti-Tg, and positive folic acid receptor blocking and binding antibody) on top of IgE-mediated autoimmunity. Further research about the cross-reactivity between IgE autoantibodies and other autoallergens may be valuable for the optimization of treatment timing and dosing (24).

The benefit of omalizumab on sleep was previously reported in urticaria (25), asthma (26), and nasal polyps (27), and on anxiety and depression in asthma (28). In the present case, we report substantial improvement in sleep disturbance and anxiety. Patients with allergic rhinitis have higher prevalence of sleep disturbance (19) and anxiety (29, 30), and most ASD patients complain of sleep disturbance or anxiety co-morbidities (31, 32). Patients with ASD and allergic rhinitis likely have even greater risk of developing these symptoms (33) that worsen brain function and general well-being.

Taken together, this case report provided new evidence for the potential efficacy of omalizumab in young children with ASD and co-morbid allergic rhinitis. By controlling IgE-mediated allergies, omalizumab improved not only allergic symptoms but also ASD-related behavioral symptoms, sleep problems, anxiety, and other neuropsychiatric symptoms. Future well-designed, randomized controlled trials are warranted in this field.

## Data Availability Statement

The original contributions presented in the study are included in the article/supplementary material, further inquiries can be directed to the corresponding author/s.

## Ethics Statement

Written informed consent to participate in this study was provided by the participants' legal guardian/next of kin. Written informed consent was obtained from the minor(s)' legal guardian/next of kin for the publication of any potentially identifiable images or data included in this article.

## Author Contributions

X-JK offered parent consultation and interviews, followed the case and collected the records, finished the first draft, and major revisions. CC and BW contributed to writing and editing. BW also finished scores and figures. All authors contributed to the article and approved the submitted version.

## Funding

This work was funded by Massachusetts General Hospital, grant number #233263.

## Conflict of Interest

The authors declare that the research was conducted in the absence of any commercial or financial relationships that could be construed as a potential conflict of interest.

## Publisher's Note

All claims expressed in this article are solely those of the authors and do not necessarily represent those of their affiliated organizations, or those of the publisher, the editors and the reviewers. Any product that may be evaluated in this article, or claim that may be made by its manufacturer, is not guaranteed or endorsed by the publisher.
